# Supplementary material for: Diagnosing Pulmonary Tuberculosis by Using Sequence-Specific Purification of Urine Cell-Free DNA
Source: J Clin Microbiol. 2021 Jul 19;59(8):e00074-21. doi: 10.1128/JCM.00074-21 (PMC8373247; doi:10.1128/JCM.00074-21)
Supplement: Supplemental file 1 — Fig. S1, Tables S1 to S4. Download JCM.00074-21-s0001.pdf, PDF file, 393 KB [file jcm.00074-21-s0001.pdf]

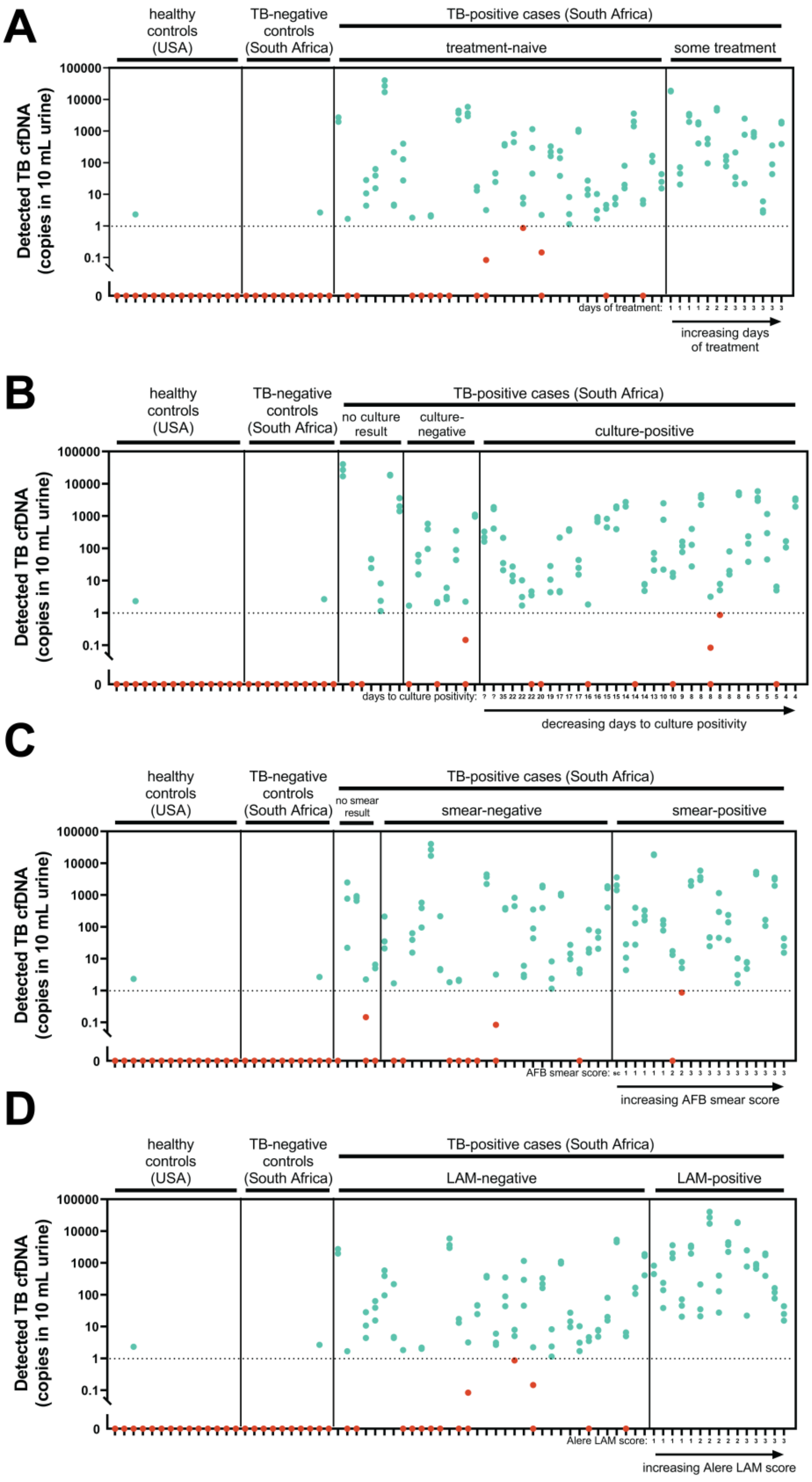

Supplemental Fig. S1: Detected TB-specific urine cfDNA concentrations. For all plots, each dot represents one of three replicates per sample, processed on different days. Cyan dots represent positive replicates with ≥1 copy detected and melting temperature matching the expected IS*6110* amplicon. Red dots represent negative replicates. At least two out of three replicates were required to be positive for a sample to be called as positive. **(A)** Stratified by treatment status and ranked by days of treatment (no correlation). **(B)** Stratified by culture result and ranked by days to culture positivity (Spearman r = -0.36 [95% CI: -0.64 to -0.0060], p = 0.041). **(C)** Stratified by smear result and ranked by smear score (no correlation). **(D)** Stratified by Alere urine LAM result and ranked by Alere urine LAM score (no correlation).

Supplemental Table S1: Comparison of sensitivities across groups.

Variable	Groups for comparison of sensitivity	P-value[a]
**HIV status**	positive vs negative	0.23
**CD4 count[b]**	≤200 vs >200 cells/mm^3	0.60
**TB treatment status**	treatment-naïve vs some treatment	0.090
**Sputum culture result**	positive vs negative	0.32
**AFB sputum smear result**	positive vs negative	0.029*
**Alere urine LAM result**	positive vs negative	0.087
**Gender**	female vs male	>0.99

* Indicates P < 0.05

[a] P-values comparing sensitivity across groups were calculated using Fisher's exact test for relative risk ratios.

[b] CD4 count was measured for HIV-positive patients only.

Supplemental Table S2: Diagnostic odds ratios indicating associations with a positive urine cfDNA result.

Variable	Comparison	Odds ratio (95% CI)[a]	P-value[b]
**HIV status**	positive vs negative	2.7 (0.68 – 10.5)	0.23
**CD4 count[c]**	≤200 vs >200 cells/mm^3	2.0 (0.28 – 13.9)	0.60
**TB treatment status**	treatment-naïve vs some treatment	infinity (0.79 – infinity)	0.090
**Sputum culture result**	positive vs negative	2.5 (0.39 – 14.7)	0.32
**AFB sputum smear result**	positive vs negative	infinity (1.6 – infinity)	0.029*
**Alere urine LAM result**	positive vs negative	infinity (1.0 – infinity)	0.087

* Indicates P < 0.05

[a] Diagnostic odds ratio of >1 indicates variables associated with higher likelihood of a positive cfDNA result. 95% confidence intervals calculated using the Baptista-Pike method.

[b] Diagnostic odds ratios were compared to a value of 1 using Fisher's exact test.

[c] CD4 count was measured for HIV-positive patients only.

Supplemental Table S3: Correlations with detected TB-specific cfDNA concentration.

Variable	Spearman's rank correlation coefficient (95% CI)	P-value
**CD4 count[a]**	-0.43 (-0.68 to -0.10)	0.011*
**Days of TB treatment[b]**	-0.36 (-0.77 to 0.26)	0.23
**Days to sputum culture positivity**	-0.36 (-0.64 to -0.0060)	0.041*
**AFB sputum smear score[c]**	-0.37 (-0.77 to 0.24)	0.21
**Alere urine LAM score[d]**	-0.094 (-0.59 to 0.45)	0.76

* Indicates P < 0.05

[a] CD4 count was measured for HIV-positive patients only.

[b] Correlation calculated only for patients with some treatment (1 – 3 days). Treatment-naïve patients were excluded.

[c] Correlation calculated only for smear-positive patients (AFB score ≥1). Smear-negative patients were excluded.

[d] Correlation calculated only for LAM-positive patients (Alere LAM score ≥1). LAM-negative patients were excluded.

Supplemental Table S4: Comparison of detected TB-specific cfDNA concentration across groups.

Variable	Groups for comparison of detected cfDNA concentration	P-value[a]
**HIV status**	positive vs negative	0.8007
**CD4 count[b]**	≤200 vs >200 cells/mm^3	0.1946
**TB treatment status**	treatment-naïve vs some treatment	0.0447*
**Sputum culture result**	positive vs negative	0.3709
**AFB sputum smear result**	positive vs negative	0.7013
**Alere urine LAM result**	positive vs negative	0.0045**
**Gender**	female vs male	0.1081

* Indicates P < 0.05; ** Indicates P < 0.01

[a] P-values calculated using Mann-Whitney test comparing sample means of cfDNA-positive samples.

[b] CD4 count was measured for HIV-positive patients only.
